# 泡腾辅助微萃取技术的开发与应用研究进展

**DOI:** 10.3724/SP.J.1123.2022.06001

**Published:** 2023-04-08

**Authors:** Hanzhang YE, Tingting LIU, Yongli DING, Jingjing GU, Yuhao LI, Qi WANG, Zhan’en ZHANG, Xuedong WANG

**Affiliations:** 1.苏州科技大学环境科学与工程学院, 江苏 苏州 215009; 1. School of Environmental Science and Engineering, Suzhou University of Science and Technology, Suzhou 215009 China; 2.江苏省环境科学与工程重点实验室, 江苏 苏州 215009; 2. Jiangsu Key Laboratory of Environmental Science and Engineering, Suzhou 215009, China

**Keywords:** 泡腾辅助微萃取, 样品前处理, 纳米材料, 离子液体, 萃取剂, 富集, 检测, effervescence-assisted microextraction (EAM), sample pretreatment, nanomaterial, ionic liquid, extractant, enrichment, detection

## Abstract

泡腾辅助微萃取(EAM)技术是一种利用CO_2_供体和H^+^供体反应产生CO_2_气泡促进萃取剂快速分散、增大与目标物的接触面积,以实现高效萃取的新型样品预处理手段。该技术具有分散速率快、萃取效率高、使用成本低、应用范围广等优点。得益于萃取剂的快速发展,泡腾辅助微萃取方法的构建和应用范围研究日趋完善和多样,已广泛用于环境、食品、生物等样品的前处理领域。该前处理技术结合各类检测仪器构建新型快速的检测方法,成功实现了重金属离子、农药、内分泌干扰物、抗生素等污染物的检测。在EAM技术的构建中,常考查泡腾片剂的组成、溶液pH、萃取温度、萃取剂种类、萃取剂添加量、洗脱剂种类、洗脱剂体积、洗脱时间、循环使用次数等因素对方法的影响,重点依据线性范围、相关系数、富集因子、检出限、定量限等参数对方法进行评判,最后结合各类仪器检测方法,实现在实际样品检测中的应用。该文从EAM技术常用的萃取剂方面入手,综述了基于纳米材料、离子液体等新兴萃取剂的EAM方法的构建,以及与液相色谱、气相色谱、原子吸收光谱或质谱等大型仪器联用,用于复杂基质中有害物质检测的研究与应用进展,分析了该技术在使用过程中存在的问题,展望了其未来在微萃取领域中的发展趋势。

## Abstract (en)

Effervescence-assisted microextraction (EAM) is a novel sample pretreatment method based on the reaction of CO_2_ and H^+^ donors to generate CO_2_ bubbles and promote rapid dispersion of the extractant. During this process, the unique dispersion method increases the contact area between the target molecule and the extraction solvent, and the adsorption/extraction efficiency of the adsorbent/extractant toward the target molecule is also enhanced. The EAM technique is of particular interest due its convenient application, low running costs, reduced solvent consumption, high extraction efficiency, and environmental friendliness. Benefiting from the rapid development of extractants, the evolution and application of the EAM technology is becoming more tuned and diversified. Indeed, the synthesis of new extractants, such as nanomaterials with multi-pore structures, large specific surface areas, and rich active sites, has attracted extensive attention, as has the development of ionic liquids with strong extraction abilities and high selectivities. As a result, the EAM technology has been widely applied to the pretreatment of target compounds in various samples, such as food, plant, biological, and environmental samples. However, since these samples often contain polysaccharides, peptides, proteins, inorganic salts, and other interfering substrates, it is necessary to remove some of these substances prior to extraction by EAM. This is commonly achieved using methods such as vortexing, centrifugation, and dilution, among others. The treated samples can then be extracted using the EAM method prior to detection using high performance liquid chromatography (HPLC), gas chromatography (GC), and atomic absorption spectroscopy (AAS) to detect substances such as heavy metal ions, pesticide residues, endocrine-disrupting compounds (EDCs), and antibiotics. Using effervescence as a novel assisted method for the dispersion of solvents or adsorbents, the concentrations of Pb^2+^, Cd^2+^, Ni^2+^, Cu^2+^, bisphenol, estrogen, and the pyrethyl pesticides have previously been successfully determined. Moreover, many influencing factors have been evaluated during method development, including the composition of the effervescent tablet, the solution pH, the extraction temperature, the type and mass/volume of extractant, the type of eluent, the eluent concentration, the elution time, and the regeneration performance. Generally, the cumbersome single factor optimization and multi-factor optimization methods are also required to determine the optimal experimental conditions. Following determination of the optimal experimental conditions, the EAM method was validated by a series of experimental parameters including the linear range, the correlation coefficient (*R*^2^), the enrichment factor (EF), the limit of detection (LOD), and the limit of quantification (LOQ). In addition, the use of this method has been demonstrated in actual sample testing, and the obtained results have compared with those achieved using similar detection systems and methods to ultimately determine the accuracy, feasibility, and superiority of the developed method. In this paper, the construction of an EAM method based on nanomaterials, ionic liquids, and other emerging extractants is reviewed, wherein the preparation method, application range, and comparison of similar extractants were evaluated for the same extraction system. In addition, the current state-of-the-art in relation to EAM research and application when combined with HPLC, cold flame AAS, and other analytical techniques is summarized in terms of the detection of harmful substances in complex matrices. More specifically, the samples evaluated herein include dairy products, honey, beverages, surface water, vegetables, blood, urine, liver, and complex botanicals. Furthermore, issues related to the application of this technology are analyzed, and its future development trend in the field of microextraction is forecasted. Finally, the application prospects of EAM in the analysis of various pollutants and components are proposed to provide reference for monitoring pollutants in food, environmental, and biological samples.

环境、食品及生物样本中有害物质的检测,能够客观地评价污染物的存在水平,也可以为评估健康风险、建立检测标准提供基础条件^[1]^。样品中复杂的基质和低的目标物含量,大大增加了仪器检测的难度。选择有效的前处理技术是同时实现降低复杂基质干扰效应、净化和富集目标污染物、准确定性定量检测的关键^[2]^。微萃取技术顺应现代检测技术绿色环保和装置小型自动化的发展趋势,成为研究的热点^[3]^。微萃取技术主要分为液相微萃取和固相微萃取,这些萃取方法拥有样品用量少、有机溶剂用量低、抗干扰能力强、萃取时间短、富集效率高和便于实现与色谱分析技术联用等优点^[4,5]^。

在这些方法中,分散方式和萃取剂对于萃取过程影响甚大。常用的分散方式有搅拌^[6]^、涡旋^[7]^、超声辅助^[8]^和泡腾辅助^[9]^等。自2011年被用作萃取过程的新型分散方式以来^[10]^,泡腾辅助微萃取(EAM)因具有便捷性和高效性被广泛应用于环境污染^[11]^、食品分析^[12]^和生物样品分析^[13]^等领域。该技术不仅避免了在萃取过程中使用大量的丙酮、乙腈等有机试剂,避免了额外使用搅拌器、涡旋仪、超声仪等仪器,甚至可以与磁性吸附剂相结合,进一步避免了离心等耗时耗力的收集方式^[14]^。样品经过洗脱后可直接使用液相色谱(LC)^[15]^、气相色谱(GC)^[16]^、原子吸收光谱(AAS)^[17]^或质谱(MS)^[18]^等仪器进行后续分析。现有报道中对液相微萃取和固相微萃取的总结与讨论非常全面,但对关于EAM技术的进展与评述却鲜有报道。因此,作者基于本课题组的研究成果,结合与该技术相关的文献报道,详细介绍了EAM技术的原理、装置、泡腾前驱体的种类、优化参数等,从纳米材料基-EAM技术、离子液体基-EAM技术、其他萃取剂基-EAM技术等方面梳理研究进展,总结该技术在食品、环境和生物样品分析中的应用,为样品前处理技术的发展提供重要的参考依据。

## 1 EAM技术介绍

EAM技术,是利用CO_2_供体、H^+^供体在溶液中充分反应产生大量的CO_2_气泡,加速萃取剂的快速分散、促使萃取剂与目标物分子充分接触,进而提高萃取效率和富集倍数的前处理技术。在该技术的构建中,常用的CO_2_供体有NaHCO_3_、Na_2_CO_3_、CH_3_COOH等,常用的H^+^供体有酒石酸(TTA)、柠檬酸、NaH_2_PO_4_等价格低廉、环境友好的试剂^[19⇓⇓-22]^。所用萃取剂主要有各类稳定性好、活性位点多的离子液体(ILs)^[23]^、碳材料^[24]^、金属化合物^[25]^、金属有机框架(MOF)^[26]^、共价有机框架(COF)^[27]^等和1-十一醇^[28]^、苯并-15-冠醚-5^[29]^、中链脂肪酸^[30]^等有机试剂。反应的容器可以是注射器^[31]^、试管^[32]^、离心管^[33]^等,适用范围较广。

依据萃取剂的不同,EAM技术可分为泡腾辅助固相微萃取和泡腾辅助液相微萃取。以本课题组^[34]^构建的泡腾辅助磁固相微萃取法为例,EAM的实验流程如图1a所示,将CO_2_供体、H^+^供体和萃取剂供体在研钵中研磨混匀,而后转移至压片机中进行压制,根据实验所需的片剂规格可选择不同型号的压片机,实际泡腾片如图1b所示。将压制好的泡腾片加入到装有样品的离心管中,CO_2_供体和H^+^供体发生泡腾反应产生的CO_2_会从底部剧烈上升,促进萃取剂材料的分散,反应通常在10 min内完成,最终形成均匀溶液。萃取剂通过离心管底部放置外部磁铁进行收集,除去上清液,沉淀用洗脱剂洗脱,收集的洗脱液在氮吹条件下干燥,然后用甲醇、乙腈等试剂回溶,回溶后过滤以除去残余的萃取剂,最后将处理后的溶液上机进行测试。将泡腾辅助用于液液分散微萃取时^[32]^,如图2a所示,操作流程基本与泡腾辅助固相微萃取类似,但是可以选择不压制泡腾片,直接分别投加CO_2_供体和H^+^供体引发泡腾反应,供体同时还可以作为萃取剂对目标物分子进行富集。实际的萃取流程如图2b所示^[35]^。

**图1:**
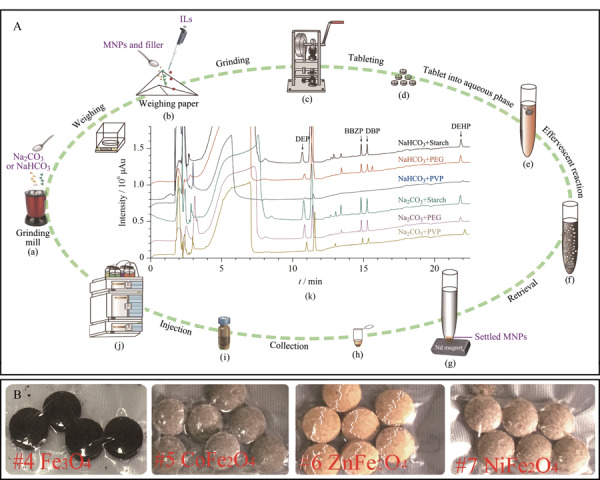
(A)泡腾辅助磁固相微萃取技术流程图和(B)实际泡腾片图^[34]^

**图2:**
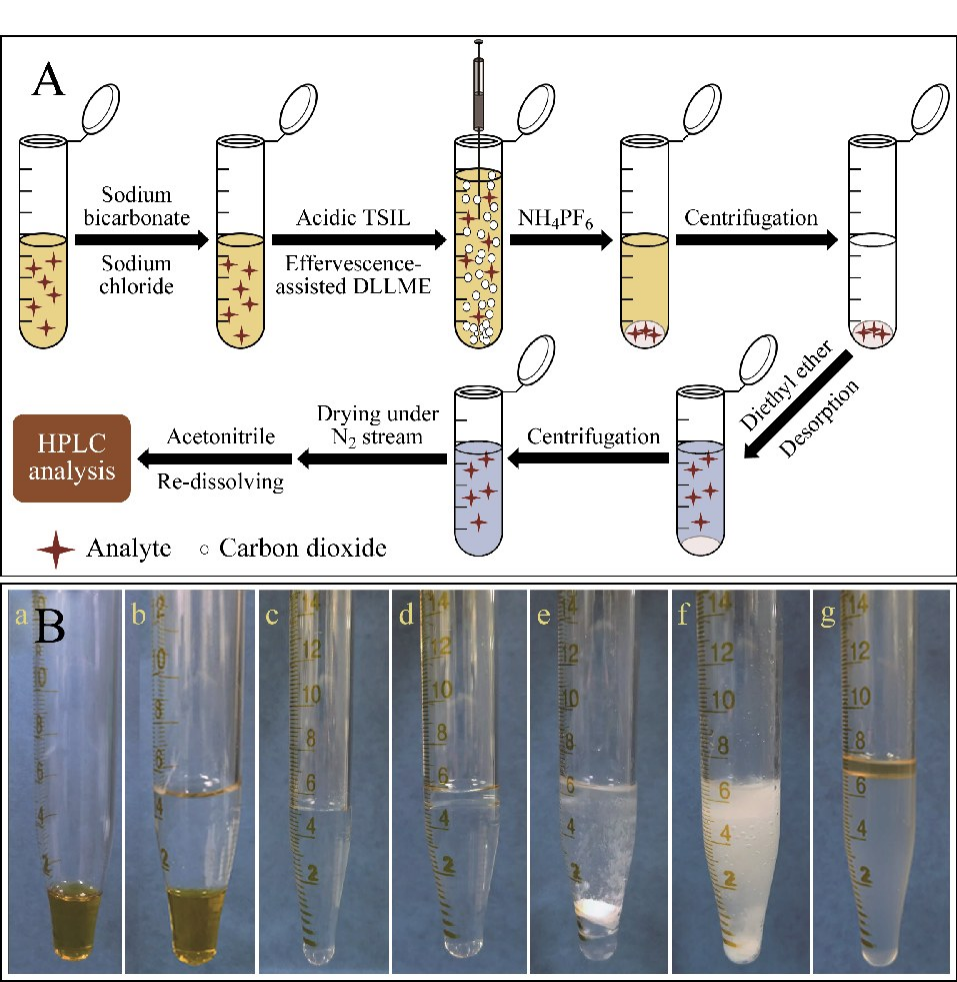
(A)泡腾辅助分散液液微萃取技术流程图^[32]^与(B)实际泡腾辅助液液微萃取流程图^[35]^

在EAM技术的应用过程中,为实现萃取通量高、耗时短、回收率高等目标,需对主要影响因子进行优化,常考查的因素有泡腾反应前驱体种类、萃取剂种类、萃取剂投加量、pH值、萃取温度、洗脱剂种类、洗脱剂体积、洗脱时间、循环使用次数等。在上述影响因子中,前驱体的组成对于泡腾反应的产生至关重要。Yang等^[33]^研究发现CO_2_和H^+^供体的选择对吸附剂的分散性和反应动力学有重要影响。实验选择NaH_2_PO_4_和柠檬酸作为H^+^供体,NaHCO_3_和Na_2_CO_3_作为CO_2_供体,结果表明:由于柠檬酸吸水性高,无论如何组合,形成的泡腾片对湿度都非常敏感,很难保持稳定;NaH_2_PO_4_和NaHCO_3_组合起泡时间过短致使吸附剂来不及完全反应;NaH_2_PO_4_和Na_2_CO_3_组合制备泡腾片反应时间、气泡速度均合适。Wang等^[23]^采用Na_2_CO_3_和NaH_2_PO_4_(1∶2,质量比)作为前驱体进行探究。当混合盐质量从200 mg增加至300 mg时,产生的CO_2_气泡逐渐增多,有效地促进了萃取剂的分散,加速了目标分析物的转移,提高了萃取效率。当混合盐质量继续增加超过350 mg时,回收率几乎保持不变,但此时溶液中盐浓度增加,溶液的离子强度和黏度均增加,使得纳米吸附剂的孔道被占据,传质效率降低,萃取性能降低。吸附剂的投加量从5 mg增加至10 mg时,得益于载体的含量越高,与目标相互作用越强,同时还促进萃取过程中的动力学,可显著提高回收率。但投加量升至10~20 mg时,萃取回收率反而下降,主要是因为纳米材料的团聚导致比表面积的降低和吸附位点的减少,对萃取过程有不利影响。

在EAM方法构建中常采用单因子优化和多因子优化结合的方式来确定最佳的实验条件。Ding等^[36]^首先对萃取剂用量、萃取时间、pH、洗脱剂种类及体积进行单因子优化。进而利用Plackett-Burman设计(PBD)从上述5个变量中筛选出影响牛奶萃取效率的重要变量(分别为pH、萃取时间和洗脱剂体积)。然后通过Box-Behnken设计(BBD)对3个重要变量进行优化,借助于Design-Expert 8.0.5软件,绘制BBD的3D响应面和等高线,以检查平均萃取回收率(ERs)与3个变量之间的关系,最终确定最佳实验条件。除上述PBD和BBD外,中心组合设计( CCD)也常用于多因子优化设计^[37,38]^。

## 2 纳米材料基-EAM技术的研究

纳米材料因具有尺寸效应、表面/界面效应等而具有良好的光学、电学、磁学等特性,其中具有比表面大、活性位点多、稳定性好的材料可作为吸附剂/萃取剂应用于分离富集、环境修复等领域。碳纳米管(CNTs)基、石墨烯基、氧化石墨烯(GO)基、金属及金属化合物基、MOF/COF基等多种纳米材料等已经被广泛用于样品前处理技术,它们可以提供多种相互作用来吸附目标污染物,进而获得令人满意的萃取能力^[39⇓⇓-42]^。吸附/萃取性能优异的纳米材料的引入,加速了技术的发展,提高了萃取效率,拓宽了应用范围。纳米材料基-EAM技术的成熟不仅可以避免有机溶剂对环境造成污染,还能通过吸附剂的改性进一步实现对目标物的高效吸附和快速分离收集的集成。基于不同纳米材料吸附剂的EAM技术的应用汇总见表1。

**表1:** 纳米材料基-EAM技术在目标物分析检测中的应用

Effervescent agents		Extractant	Analytes	Samples	Sample preparation	Measurement	Extraction time	Recoveries/ %	Ref.
CO_2_ source	H^+^ source								
Na_2_CO_3_	NaH_2_PO_4_	MWCNTs	triazines	water	MWCNT	UPLC-UV	3 min	77-101	[11]
Na_2_CO_3_	NaH_2_PO_4_	NiFe_2_O_4_ MNPs	heavy metals	seafood extracts	MNET-DSPM	ICP-MS	< 3 min	73.4-99.3	[12]
Na_2_CO_3_	NaH_2_PO_4_	GMWCNTs-COOH	natural antioxi- dants	hawthorn herb	ENMAM	UHPLC-ECD	10 min	15-96	[21]
Na_2_CO_3_	citric acid	nano-(Fe_3_O_4_/CS-Se)_2_	heavy metals	sausage extracts and water	EA-DM-μSPE	μS-FAAs	4.5 min	95.6-104.1	[22]
NaHCO_3_	NaH_2_PO_4_	sulfonate PS-DVB- CNT	alkaloids and flavonoids	biological samples	EPT-SPME	UHPLC-UV	1.2 min	90.05-99.85	[24]
Na_2_CO_3_	citric acid	Fe_3_O_4_@SiO_2_@N_3_ MNPs	antidepressant drugs	urine and pharma- ceutical wastewater	EA-DM-mSPE	/	< 6 min	≥70	[25]
Na_2_CO_3_	NaH_2_PO_4_	core-shell mag- netic COF	endocrine dis- ruptors	water, beverages and biosamples	MNER-EM	HPLC-FLD	5 min	83.4-106.2	[27]
Na_2_CO_3_	NaH_2_PO_4_	mesoporous hy- brid materials (PCMA-60)	tanshinones	root extracts (CDDP, DT)	DMSPE	UPLC	30 min	90-101	[31]
Na_2_CO_3_	NaH_2_PO_4_	β-cyclodextrin/attapulgite compos- ite	pyrethroids	water	EAIS-DSPE	HPLC-UV	-	76.8-86.5	[33]
Na_2_CO_3_	tartaric acid	mETDSE/Ni@ N-GrTs	trace bisphe- nols	milk	mETDSE	HPLC-FLD	6.3 min	83.6-105.1	[36]
Na_2_CO_3_	NaH_2_PO_4_	ATP/PPY/Fe_3_O_4_	pyrethroids	honey	MEA-DSPE	HPLC-DAD	5 min	81.42-106.73	[43]
NaHCO_3_	citric acid	Fe_3_O_4_@porous activated carbon	phenolic endo- crine disrup- ting chemicals	environmental water	META-DES- DLLME	HPLC	30 s	81.0-94.7	[44]
Na_2_CO_3_	tartaric acid	mNH_2_-MIL-101 (Al)@β-CD@GO	PAHs and BPs	roasted meat	ENCG	HPLC-FLD	40 s	86.9-103.9	[45]
Na_2_CO_3_	tartaric acid	Ni@N-CNTs	estrogens	milk	MSPE	HPLC-FLD	3 min	85.2-102.9	[46]
Na_2_CO_3_	citric acid	graphene oxide modified with dopamine	metal ions	sausages and water	EA-DM-mSPE	ASS	3 min	95.5-98.0	[47]

MWCNTs: multi-walled carbon nano-tubes; MNPs: magnetic nanoparticles; MNET-DSPM: effervescent tablet-assisted dispersive solid-phase microextraction; ICP-MS: inductively coupled plasma mass spectrometry; GMWCNTs-COOH: carboxyl graphitized multi-walled carbon nanotubes; ENMAM: effervescence and nylon membrane assisted microextraction; UHPLC-ECD: ultrahigh performance liquid chromatography-electrochemical detection; EA-DM-μSPE: effervescent salt-assisted dispersive magnetic micro solid-phase extraction; μS-FAAs: micro sampling flame atomic adsorption spectroscopy; PS-DVB-CNT: sulfonate carbon nanotube/polystyrene-divinylbenzene composite; EPT-SPME: effervescent pipette tip solid phase microextraction; EA-DM-mSPE: effervescent-assisted dispersive magnetic micro solid-phase extraction; MNER-EM: magnetic NiFe_2_O_4_@COF-based effervescent reaction-enhanced microextraction; HPLC-FLD: high-performance liquid chromatography-fluorescence detection; DMSPE: effervescence-assisted dispersive micro-solid-phase extraction; EAIS-DSPE: effervescence-assisted *β*-cyclodextrin/attapulgite composite for the in-syringe dispersive solid-phase extraction; mETDSE/Ni@N-GrTs: effervescent reaction-assisted dispersive solid-phase extraction/Ni-based N-doped graphene tubes; ATP/PPY/Fe_3_O_4_: magnetic attapulgite/polypyrrole sorbent; MEA-DSPE: magnetic effervescence-assisted dispersive solid-phase extraction; META-DES-DLLME: effervescent tablet-assisted deep eutectic solvent-based dispersive liquid-liquid microextraction; ENCG: effervescence-enhanced dispersion, the mNH_2_-MIL-101(Al)@*β*-CD@GO-based adsorption/extraction; HPLC-DAD: high-performance liquid chromatography-diode array detection; ASS: adsorption spectroscopy. CDDP: compound Danshen dripping; DT: Danqi tablet; PAHs: polycyclic aromatic hydrocarbons; BPs: bisphenolic pollutants.

### 2.1 无磁性纳米材料基

Lasarte-Aragonés等^[10]^首次将商业化吸附剂Oasis HLB与NaH_2_PO_4_、Na_2_CO_3_混合制备出泡腾片用于固相微萃取过程,通过研究对比发现,泡腾辅助的分散方式能耗小、操作便捷、对萃取效果提升明显。泡腾片可提前制备,便于保存和运输,大大缩短了样品预处理的时间,具有较大的污染物现场提取的应用潜力。对于基于商业化吸附剂构建的方法而言,萃取效率的提高主要依赖于泡腾辅助分散方式的高效率和吸附剂本身优异的物理吸附性能。如需进一步提高EAM方法的萃取效率,需要探索出更多性能优异的吸附剂。Ye等^[31]^采用简单的溶剂热法制备了具有大比表面积和高度有序孔道结构的介孔杂化材料PCMA-60,用作EAM技术的萃取剂,成功用于提取复杂植物制剂中微量成分。Yang等^[33]^首次构建了以*β*-环糊精/凹凸棒土复合物(*β*-CD/ATP)为吸附剂/萃取剂的EAM技术。*β*-CD是一种表面亲水、内部有疏水腔的环状低聚糖,可以通过主-客体作用(主要是疏水作用)与某些分子形成包合物,具有分子识别效应;ATP为一种晶质水合镁铝硅酸盐矿物,具有独特的层链状结构特征,在其结构中存在晶格置换,晶体呈针状、纤维状或纤维集合状,具有较高的吸附能力。得益于单体的独特性质和复合材料成分间的协同作用,该吸附剂在环境水样中拟除虫菊酯的富集中表现优异。此外,ATP/PPY/Fe_3_O_4_^[43]^、mNH_2_-MIL-101(Al)@*β*-CD@GO^[45]^、IL-M-*β*-CD/ATP^[15]^等不同修饰的复合材料也成功用于EAM技术中。

除与ATP、*β*-CD等化合物复合外,与碳纳米材料复合也是研究者们常用的提升吸附/萃取能力的改性手段。Wang等^[21]^首次将石墨化碳纳米管(GMWCNTs)用作EAM技术的吸附剂用于提取山楂样本中的抗氧化酚类物质。GMWCNTs为在惰性气体下热处理(2800 ℃)约20 h得到的纯化CNTs,纯度高达99.99%,粉体结晶程度高,具有良好的导电、导热和力学性能。得益于GMWCNTs独特的结构和大比表面积,在山楂样品前处理中得到了高萃取回收率。Wang等^[24]^将CNT加入磺化聚苯乙烯-二乙烯基苯微球中制备新型复合型聚合物吸附剂(sulfonate PS-DVB-CNT)。利用Langmuir和Freundlich模型比较分析sulfonate PS-DVB和sulfonate PS-DVB-CNT两种吸附剂对淫羊藿中生物碱和黄酮类化合物的提取过程发现,sulfonate PS-DVB-CNT的吸附容量约为113.6~163.9 mg/g,远高于sulfonate PS-DVB的吸附容量99.0~114.9 mg/g。在吸附剂的形成过程中,聚合物颗粒负载在碳管表面,可有效增大比表面积,进而增加与目标物分子的接触面积和接触位点,使得sulfonate PS-DVB-CNT在萃取过程中表现出良好的吸附能力、脱附效率和亲水性。另外该工作省略了压制泡腾片的步骤,直接采用混匀吸附剂、NaHCO_3_、NaH_2_PO_4_粉末后加入溶液中进行泡腾反应的方式进行提取,进一步缩短了预处理时间,但由于粉末态供体不易携带且难以在野外准确称量,该方法仅适用于实验室条件。

### 2.2 磁性纳米材料基

吸附剂的创新及泡腾反应方式的多样性进一步完善了EAM技术。在上述基于非磁性纳米材料基-EAM技术中,对萃取剂的离心收集成为预处理过程中的耗时步骤。为克服该缺点,使样品前处理效率更高,研究者们致力于对萃取剂进行磁化修饰,常用的磁核有Fe_3_O_4_^[43]^、NiFe_2_O_4_^[48]^、Fe_3_S_4_^[19]^、金属镍颗粒^[36,46]^等。Fahimirad等^[22]^在磁性Fe_3_O_4_-壳聚糖复合材料表面接枝二苯基二硒烯,形成一种新型磁性纳米吸附剂(Fe_3_O_4_/CS-Se)_2_。该吸附剂材料最大饱和磁通量约为24.65 emu/g,可使用外部磁铁进行快速分离。其中,磁性Fe_3_O_4_纳米颗粒有两个重要用途:(1)便于吸附剂从样品溶液中分离;(2)与壳聚糖吸附剂结合,增大吸附剂材料的接触面积,进而增强对目标物的吸附作用。该方法用于水样中Pb(Ⅱ)、Cd(Ⅱ)、Ni(Ⅱ)和Cu(Ⅱ)离子的富集与提取,萃取时间缩短至4.5 min。Fahimirad等^[25]^基于纳米材料改性策略,引入富氮官能团进行修饰,合成了一种新型磁性Fe_3_O_4_@SiO_2_@N_3_纳米复合材料吸附剂。该吸附剂除拥有良好的磁性外,表面的富氮基团提供了更强吸附力和更多吸附位点,大大增加了材料表面结合污染物分子的能力。但在影响因素优化中发现,当固定Na_2_CO_3_用量为100 mg时,柠檬酸含量高于150 mg会明显降低溶液pH,影响吸附剂中磁核的稳定性。

在磁核的引入过程中,分散性差是急需解决的重要问题,研究者们选择将磁核与拥有大比表面积的石墨烯、MOF、COF等材料进行复合来避免其团聚。Liu等^[45]^结合GO具有大的*π*-*π*共轭体系、NH_2_-MOF对中等强度极性污染物具有强的吸附能力、*β*-CD具有识别目标物能力等优势,以NiFe_2_O_4_为磁核制备了mNH_2_-MIL-101(Al)@*β*-CD@GO复合材料。将该材料用作EAM技术中的萃取剂,在宽极性污染物(lg *k*_ow_=3~6)范围内有效的同时提取了多环芳烃类污染物(PAHs)和双酚类化合物(BPs),这为多功能复合吸附剂的制备与应用提供了借鉴。在方法的构建过程中探究了磁核与不同的前驱体材料复合顺序对测试结果的影响,结果发现先对GO进行磁化形成mGO时,回收率仅有20%~32%;而优先形成磁性NH_2_-MOF前驱体时,回收率提高至41%~73%。不同的改性方式决定着吸附/萃取剂表面暴露出的活性位点多少,对萃取过程有着重要的影响。Tan等^[27]^以NiFe_2_O_4_为磁核,以COF材料为壳制备了具有较大比表面积(169.7 m^2^/g)的NiFe_2_O_4_@COF复合材料作为吸附剂。COF材料作为磁核的包覆层,比MOF材料拥有更好的热稳定性和化学稳定性,使得该复合材料在酸性介质和有机溶液中的可再生性能更强。作者首次将该磁性功能化的COF纳米材料用于EAM技术中,对比不同的磁核前驱体发现萃取效率的大小关系为NiFe_2_O_4_@COF>Fe_3_S_4_@COF>Fe_3_O_4_@COF>NiFe_2_O_4_。在最优条件下,表面包裹COF后,磁核溶解在溶液中的铁离子仅占最大理论质量浓度的0.020%~0.086%,对萃取内分泌干扰物(EDs)几乎没有影响,这也为磁核的保护提供了借鉴。

在包覆磁核的过程中,包覆均匀度、包覆层厚度对萃取剂的磁性能和萃取性能影响较大。为同时获得包覆均匀、比表面积大且制备方法简便的优异磁性萃取剂,Ding等^[36]^采用高温煅烧法一步制备了磁性碳纳米材料(Ni@N-GrTs),将磁核金属镍颗粒直接包裹于碳材料中,且实现了氮元素的一步掺杂,这不但提高了磁核的稳定性,还克服了传统碳材料的反应位点少的问题。将制备好的Ni@N-GrTs与TTA、Na_2_CO_3_混合压制成泡腾片剂,构建了同时集高效吸附提取、分散、磁分离收集于一体的EAM技术,避免了传统分散方式的实验室局限性和化学污染性。通过对吸附机理探究发现,掺杂的N原子以吡啶氮基团的形式存在于Ni-N-GrTs的结构中,缺陷位点的增加、*π-π*共轭和氢键作用的增强,使得吸附剂对于BPs分子的吸附能力大大增强。这也为磁核保护和掺杂型碳纳米复合材料用于EAM技术提供了新的思路。

## 3 离子液体基-EAM技术的研究

离子液体是完全由离子组成的有机盐,在室温或接近室温时是液体,对多种有机物具有优异的溶解能力,且自身无毒无污染、蒸汽压低、液程宽、电化学稳定性好,还可通过各种相互作用与各种分子结合,如*π-π*、氢键、静电、色散和偶极相互作用等,所以被认为是理想的萃取溶剂或萃取剂^[49⇓-51]^。常见ILs中所用到的阴离子有Cl^-^、Br^-^、I^-^、[NTF_2_]^-^、[H_2_PO_4_]^-^、[BF_4_]^-^、[PF_6_]^-^等,阳离子主要有咪唑类、吡啶类、吡咯类、哌啶类、四烷基铵类和四烷基膦类等^[52,53]^。将不同的阴阳离子进行配对,理论上可组成的ILs种类高达1万亿种,通过调节阴离子和阳离子的组成,可实现调整其吸附性能和吸附选择性的目的^[54]^。ILs的特殊性质决定了其主要应用于萃取分离^[55]^、化学催化^[56]^和电化学^[57]^等。其中在萃取分离的过程中,ILs可用作液液萃取过程中的萃取剂,以替代传统的有机试剂,实现萃取过程的“绿色化”^[58]^。相比于有机溶剂,ILs作为萃取剂可以提高测定方法的灵敏度、选择性和准确性。但纯ILs本身黏度大,在萃取过程中存在流失严重、传质效率低、重复使用效率低、与目标物分离困难等问题^[59]^。

为解决上述问题,研究者们致力于通过物理吸附或化学键合的方式,将ILs固载于多孔材料、高分子材料、磁性纳米材料、分子印迹材料等固体表面,制备成复合型固相萃取剂,使吸附剂兼具ILs和原固体材料的综合特性,可用于重金属离子、内分泌干扰物、农药等有害物质的检测^[60]^,还可作为选择性吸附剂直接从天然植物中提取某些活性成分^[55]^。

在EAM技术中,ILs常用作萃取剂或萃取剂的修饰层。不同ILs的碳链长度、黏度和水溶性均不同,对萃取效率的影响也不尽相同(如表2所示)。Yang等^[37]^将磁回收技术与ILs-EAM技术相结合,使萃取剂的分散和收集几乎同时完成。实验考查了[HMIM]Cl、[OMIM]Cl、[HMIM]PF_6_、[OMIM]PF_6_、[HMIM]NTF_2_和[OMIM]NTF_2_的使用效果,其中[OMIM]PF_6_不易与磁性纳米颗粒复合,[OMIM]NTF_2_影响偶氮菌素的检测。[HMIM]Cl和[OMIM]Cl在水中的溶解度相对较高,难以收集,萃取效果不理想,不建议选择为萃取剂。Wang等^[17]^考查了基于磁性离子液体[C_4_MIM][FeCl_4_]、[C_6_MIM][FeCl_4_]和[C_8_MIM][FeCl_4_]的EAM技术用于天然蔬菜中微量无机砷的萃取性能。[C_4_MIM][FeCl_4_]萃取回收率最高,另外两种ILs回收率较低的主要原因可能是较长的烷烃链、较高的密度和黏度使得它们在吸附后较难通过外部磁体从水溶液中回收。Li等^[19]^采用磁性更强、比表面积更大的Fe_3_S_4_纳米颗粒作为磁核,考察了[C_4_MIM]PF_6_、[C_6_MIM]PF_6_、[C_8_MIM]PF_6_ 3种离子液体用于萃取6种多溴联苯醚(PBDEs)的效果。[C_4_MIM]PF_6_黏度低、溶解性高有利于分散在水溶液中进而促进萃取效率的提高,[C_8_MIM]PF_6_的水溶性低、黏度大,在水中不能完全分散,不利于样品的萃取,因此在加入[C_4_MIM]PF_6_时,PBDEs的回收率最高,约为82.6%~97.3%。

**表2:** 离子液体基-EAM技术在目标物分析检测中的应用

Effervescent agents		Extractant	ILs type	Analytes	Samples	Sample preparation	Measurement	Extraction time/min	Recoveries/ %	Ref.
CO_2_ source	H^+^ source									
Na_2_CO_3_	TTA	[C_6_MIM]PF_6_	[C_6_MIM]BF_4_	endogenous steroids	serum and urine	IS-META-ILDM	HPLC-UV	3	90.0-118.5	[13]
Na_2_CO_3_	NaH_2_PO_4_	IL-M-β-CD/ATP	[OMIM]NTF_2_	fungicides	honey/juice	EA-DSPE	HPLC-DAD	3	77.0-94.3	[15]
Na_2_CO_3_	HCl	[C_6_MIM]PF_6_	[C_6_MIM]PF_6_	pyrethroids	milk	MET-ILM	GC-ECD	2	78.3-101.8	[16]
Na_2_CO_3_	NaH_2_PO_4_	[C_4_MIM][FeCl_4_]	[C_4_MIM][FeCl_4_]	As	vegetable	ETA-MILs-ME	GFAAS	<1	97.9-105.8	[17]
Na_2_CO_3_	NaH_2_PO_4_	IL@SiO_2_@Fe_3_O_4_	[BMIM]PF_6_	beta block- ers	plasma	DSPE-IL@MNP-EP	LC-MS		75-91	[18]
Na_2_CO_3_	NaH_2_PO_4_	[C_4_MIM]PF_6_	[C_4_MIM]PF_6_	PBDEs	water/milk/ serum	META-IL-DLLME	HPLC-DAD	<1	77.3-106.7	[19]
NaHCO_3_	TTA	[BMIM]PF_6_	[BMIM]PF_6_	BUIs	fruit juice/ vegetable	EA-DLLME	HPLC-DAD	6	74-93	[20]
Na_2_CO_3_	NaH_2_PO_4_	[C_6_MIM]PF_6_	[C_6_MIM]PF_6_	Se	beverage/food	MEA-IL-DLLME	GFAAS		92.0-108.1	[23]
Na_2_CO_3_	NaH_2_PO_4_	[C_6_MIM]PF_6_	[C_6_MIM]PF_6_	PAHs	milk	NIE-DSM	HPLC-FLD	3	74.1-101.6	[26]
NaHCO_3_	acidic TSIL	[C_4_MIM][HSO_4_]	[C_4_MIM][HSO_4_]	triazine her- bicides	tea beverage	EA-DLLME	HPLC-UV	2	77.1-114.7	[32]
Na_2_CO_3_	starch	[HMIM]NTF@ NiFe_2_O_4_	[HMIM]NTF	phthalate esters	milks	MMIL-EFM	HPLC-UV	7	94.8-105.6	[34]
Na_2_CO_3_	LPIL	tetradecyl(tri- hexyl) phospho- nium chloride	CYPHOS IL 101	PAHs	edible oils	EM-LPSH	HPLC-FLD	5	80.1-103.3	[35]
Na_2_CO_3_	NaH_2_PO_4_	[HMIM]NTF_2_	[HMIM]NTF_2_	fungicide	water	META-IL-DLLME	HPLC-DAD	1	70.7-105	[37]
Na_2_CO_3_	NaH_2_PO_4_	[C_4_(MIM)_2_] [N(CN)_2_]	[C_4_(MIM)_2_]Br_2_	PAHs	meat	EIDLM	HPLC-FLD	4	82.3-104.7	[48]
Na_2_CO_3_	NaH_2_PO_4_	IL-TiO_2_ nanofluid	[OMIM]NTF_2_	acaricide	honey and tea	EA-DLLME	HPLC-DAD	5	64.08-92.65	[59]

TTA: tartaric acid; IS-META-ILDM: magnetic effervescence tablet-assisted microextraction coupled to in situ metathesis reaction of ionic liquid; MET-ILM: magnetic effervescent tablet containing ionic liquid microextraction; ECD: electron capture detector; ETA-MILs-ME: effervescence tablet-assisted magnetic ionic liquids-based microextraction; GFAAS: graphite furnace atomic absorption spectrometry; IL: ionic liquid; MNP: magnetic nanoparticles (SiO_2_@Fe_3_O_4_); EP: effervescent powder; PBDEs: polybrominated diphenyl ethers; META-IL-DLLME: magnetic effervescent tablet-assisted ionic liquid-based dispersive liquid-liquid microextraction; BUIs: benzoylurea insecticides; EA-DLLME: effervescence assisted-dispersive liquid-liquid microextraction; NIE-DSM: multi-layer core-shell nanocomposites and ILs-based effervescence-assisted dispersive solid phase microextraction; TSIL: task-specific ionic liquid; MMIL-EFM: MFe_2_O_4_-based magnetic ionic liquid effervescent tablet microextraction (M=Co, Ni, Zn); LPIL: lighter-than-water phosphonium-based ionic liquids; EM-LPSH: effervescent-assisted dual microextraction based on LPILs and switchable hydrophilic/hydrophobic fatty acids; EIDLM: effervescence-enhanced in situ dispersive liquid-liquid microextraction based on imidazolium-based dicationic ionic liquids and NiFe_2_O_4_.

在ILs基-EAM技术中,少量ILs不足以充分萃取目标分子;过量ILs则会由于体系黏度过高致使在压片过程中损失更多的萃取剂,因此,常需考察不同体积的ILs对萃取效果的影响。Zhou等^[26]^考查[C_6_MIM]PF_6_加入体积为10~60 μL时回收率的变化,最终选择50 μL作为最佳投加量。Wu等^[60]^制备了ILs-TiO_2_纳米流体作为萃取剂,对杀螨剂进行萃取。将TiO_2_纳米颗粒以0.05%、0.1%、0.2%、0.4%的质量分数分散在[OMIM]NTF_2_中作为提取溶剂,结果表明,0.2% (质量分数) [OMIM]NTF_2_基纳米流体对杀螨剂的提取效率最佳。在20~60 μL范围内,考查目标物的回收率随萃取剂体积的变化。当萃取剂体积从20 μL增加至30 μL时,回收率随之逐渐增加;当萃取剂体积大于30 μL时,萃取效率有较大地下降,这可能是由于加入萃取剂越多,制备泡腾片过程中损失会越严重,反而对方法造成不利影响。ILs对于温度的变化也十分敏感,适宜的温度可以使ILs在水相中分散得更好,可以进一步增大水相与ILs间的接触面积,有利于更快地萃取出更多的待测物。另外,温度过低会减慢CO_2_的生成速度,导致泡腾分散的效果变差;温度过高,会增强布朗运动,加快体系的传质速率,缩短待测物与萃取剂的接触时间,降低萃取效率。因此,选择合适的萃取温度对于萃取体系的构建同样不可或缺。

## 4 其他萃取剂基泡腾辅助萃取技术

除纳米材料、ILs类物质可用于EAM技术外,研究者们还开发了其他类型萃取剂基预处理方法(如表3所示)。Liu等^[28]^采用1-十一醇作为萃取溶剂,结合EAM技术构建了基于黏性浮动有机液滴的EA-DLLME技术,在萃取过程中,1-十一醇会在水相中形成大液滴漂浮在表面,泡腾反应产生的CO_2_气泡会强力冲散1-十一醇大液滴形成小液滴,待CO_2_气泡消失,萃取了目标分子的小液滴重新凝聚,通过移液器收集即可。Gao等^[61]^将泡腾辅助与固化悬浮有机液滴相结合构建了基于可转换脂肪酸的微萃取技术,成功用于快速测定海水、沉积物和海产品中氟喹诺酮类药物和四环素。将5种中链脂肪酸(戊酸、己酸、庚酸、辛酸和壬酸)作为萃取溶剂,依据它们在不同pH条件下亲/疏水形态转换的能力强弱,比较萃取效果的差异发现,壬酸对6种抗生素的提取回收率最高(>92%),且在低温下能由液体变为凝固漂浮状态,因此选择壬酸作为最佳提取溶剂。该方法有以下突出优点:(1)盐与脂肪酸的反应过程使萃取溶剂由疏水状态变为亲水状态;(2)CO_2_气泡大大增加了脂肪酸与分析物之间的接触面积,从而提高了萃取回收率;(3)脂肪酸在低温下凝固,便于分离,避免了使用专用设备。除上述基于1-十一醇的黏性悬浮有机液滴与基于壬酸的可转换试剂外,其他类可转换亲水性溶剂^[62]^、冠醚类化合物^[29]^、深共熔溶剂^[67]^等均成功用于EAM技术中,并取得了良好的富集效果。

**表3:** 其他吸附剂基-EAM技术在目标物分析检测中的应用

Effervescent agents		Extractant	Analytes	Samples	Sample preparation	Measurement	Extraction time/min	Recoveries/ %	Ref.
CO_2_ source	H^+^ source								
Na_2_CO_3_	citric acid	1-undecanol	triazine herbicides and triazole fungi- cides	water	EA-DLLME-CFO	LC-MS	3	72.4-101.5	[28]
NaHCO_3_	NaH_2_PO_4_	sodium dodecyl sulfate	coumarins	cortex fraxini	EA-MSPD	UHPLC	5	91.37-100.29	[29]
Na_2_CO_3_	H_2_SO_4_	hexanoic acid	toxic azo dyes	food	CO_2_-EA-EME-SS	HPLC	1	86.6-104.5	[30]
Na_2_CO_3_	fatty acid	nonionic acid	antibiotics	seawater, sedi- ment, and seafood	EA-SFAM-SFO	HPLC-UV	5	82.2-116.7	[61]
NaHCO_3_	CH_3_COOH	hydrophobic deep eutectic solvent	colorants	food	EA-DLLME-DES	UV-Vis spec- troscopy	10	97.9-101.7	[62]
NaHCO_3_	citric acid	octanoic acid	endocrine disrup- tor	bottled beverages	ETA-SHS-ME-SFO	HPLC	1.5	71.7-98.1	[63]
NaHCO_3_	citric acid	Fe_3_O_4_	triazine herbicides	water	META-SHS-LPME	HPLC	5	81.4-96.7	[64]
Na_2_CO_3_	formic acid	hydrophobic deep eutectic solvent	nonsteroidal anti- inflammatory drugs	liver	EA-DLLME	HPLC-MS/MS	5	92-108	[65]
NaHCO_3_	citric acid	hydrophobic deep eutectic solvent	strobilurin fungi- cides	water, juice, wine, and vinegar	ETA-ME-SDES	HPLC	5	77.4-106.9	[66]

EA-DLLME-CFO: effervescence assisted dispersive liquid-liquid microextraction based on cohesive floating organic drop; EA-MSPD: effervescence assisted-matrix solid-phase dispersion; CO_2_-EA-EME-SS: effervescence assisted emulsification microextraction method using an efficient switchable solvent; EA-SFAM-SFO: effervescence-assisted switchable fatty acid-based microextraction combined with solidification of a floating organic-droplet; EA-DLLME-DES: effervescence assisted dispersive liquid-liquid microextraction based on a hydrophobic deep eutectic solvent; ETA-SHS-ME-SFO: effervescence tablet-assisted switchable solvent-based microextraction- solidification of floating organic droplets; META-SHS-LPME: magnetic effervescence tablet-assisted switchable hydrophilicity solvent-based liquid phase microextraction procedure; ETA-ME-SDES: effervescence tablet-assisted microextraction method based on the solidification of deep eutectic solvent.

## 5 EAM技术在复杂基质中的应用

EAM技术结合多种分析仪器用于污染物的检测在食品安全、环境监测、药物分析等领域得到了广泛的应用。

### 5.1 食品样品

近年来,随着我国食品行业科学技术的飞速发展以及民众越来越高的食品安全意识,食品安全检测的需求越来越大。食品基质通常成分复杂,干扰组分多,稳定性差,有机污染及重金属污染物含量更低甚至痕量,食品基质的安全分析是复杂基质中的痕量分析^[68]^。传统的食品基质的预处理方法(如固相微萃取、液相微萃取、共沉淀、DLLME等)有一些缺点,如处理时间长、富集因子不理想、需使用大量有机溶剂、可能造成二次污染等^[69]^。近年来,EAM技术的发展,突破了传统食品行业检测技术的瓶颈,成功应用于奶制品、蜂蜜、果汁、蔬菜、香肠和肉类等样品检测领域。

Zhou等^[16]^将EAM技术与气相色谱-电子捕获检测器(GC-ECD)检测技术联用用于检测牛奶中的5种拟除虫菊酯(联苯菊酯、甲氰菊酯、苄氯菊酯、溴氰菊酯和氰戊菊酯),采用最优条件,在实际牛奶样品(全脂、半脱脂和脱脂)检测中,5种拟除虫菊酯的检出限为0.024~0.075 μg/kg,回收率为78.3%~101.8%,日内和日间回收率的相对标准偏差(RSD)分别为<4.8%和<6.3%。相较于液液萃取-低温冷冻(LLE-Freezer)-GC-ECD (0.25~0.75 μg/kg)^[69]^,液液萃取-硅藻土(LLE-Florisil)-GC-ECD (15~60 μg/kg)^[70]^和QuEChERS-GC-ECD (0.1~0.8 μg/kg)^[71]^而言,新的磁性泡腾辅助-离子液体基微萃取(MET-ILM)方法降低了拟除虫菊酯的检出限,体现了该方法在食品和环境检测方面具有的巨大应用潜力。继而,他们通过合成新型吸附剂NiFe_2_O_4_@SiO_2_@PVP@NH_2_-MIL-101(Fe)用于EAM技术^[26]^,成功用于牛奶中5种PAHs的富集。Ding等^[36]^将EAM技术与HPLC-FLD仪器设备联用构建mETDSM/Ni@N-GrTs方法用于检测奶制品中的BPA、BPB、BPAF和BPAP,方法的检出限为0.1~0.2 μg/L,日内和日间回收率的RSD分别为1.9%~4.9%和2.5%~6.8%。在全脂、低脂和脱脂牛奶以及奶茶和婴儿奶粉中,平均提取回收率在83.6%~105.1%之间,RSD为0.9%~4.7%。

EAM技术在蜂蜜、果汁等液体样品检测领域同样受到了关注。Wu等^[15]^将EAM技术与HPLC-DAD结合构建了基于离子液体改性磁性-*β*-环糊精/凹凸棒石为萃取剂的泡腾辅助-分散固相萃取(IL-M-*β*-CD/ATP EA-DSPE)体系用于提取和预浓缩蜂蜜和果汁中的嘧菌酯、三唑酮、嘧菌环胺和肟菌酯。该方法的提取效率和精密度高于其他用于相同基质的杀菌剂检测方法。Bamorowat等^[20]^将泡腾辅助与超声辅助技术联用用于DLLME过程,结合HPLC-DAD检测,用于提取和富集果汁和蔬菜样品中的苯甲酰脲类杀虫剂(六氟脲、特氟隆、三氟脲和氯氟脲)。在最佳条件下,检出限和定量限分别为0.04~0.19 ng/mL和0.13~0.64 ng/mL,富集因子和萃取回收率分别为370~465和74%~93%,在卷心菜样品中检出了六氟脲,含量为(94±6) ng/g。Yang等^[43]^将EAM与HPLC-DAD技术联用,成功用于蜂蜜样品中5种拟除虫菊酯的检测。Wang等^[23]^建立了磁性泡腾辅助离子液体分散液液微萃取-石墨炉原子吸收法(MEA-IL-DLLME-GFAAS)测定食品饮料样品中硒形态的新方法,方法应用于红茶、奶粉、蘑菇、大豆、竹笋、能量饮料、瓶装水、碳酸饮料和矿泉水等食品饮料样品中Se(Ⅳ)和Se(Ⅵ)的形态测定,相对回收率为92.0%~108.1%。Piao等^[32]^建立了一种简便、快速、灵敏、环保的EAM与HPLC联用技术测定茶饮料中三嗪类除草剂的含量,在实际样品中除草剂的回收率在76.3%~135.9%之间。Wu等^[60]^建立了一种简单实用的离子液体基TiO_2_纳米流体泡腾辅助分散液液微萃取(EA-DLLME)-HPLC-DAD用于检测蜂蜜和茶叶中杀螨剂,该方法具有提取时间短、回收率高、富集系数高、有机溶剂消耗少、操作简便等优点,可广泛用于蜂蜜和茶叶样品中农药的测定。

除液态样品外,EAM技术在香肠、肉类等固体食品样品的检测领域同样得到了成功应用。Zhang等^[48]^在最优条件下,将EAM技术与HPLC-FLD结合,用于在生肉、煮熟肉、炭烤肉和烟熏肉中5种多环芳烃(芴(FLU)、蒽(ANT)、芘(PYR)、䓛(CHR)和苯并芘(BaP))的检测。方法的检出限低至0.01~0.07 μg/kg,回收率高达82.3%~104.7%。Fahimirad等^[22]^将EAM与微火焰原子吸收光谱法(MAAS)联用,实现了香肠中的Pb(Ⅱ)、Cd(Ⅱ)、Ni(Ⅱ)、Cu(Ⅱ)的定性定量检测。Zhou等^[12]^将EAM和ICP-MS技术联用,成功实现了海产品中Mn、Cu、Zn、Cd离子的检测。Liu等^[45]^将EAM技术与HPLC-FLD技术联用,成功用于同时检测5种烤肉样品中的BPA、BPB、BPF、BPAP、PHE、CHR和PYR含量。

### 5.2 环境样品

EAM技术在环境基质中的应用主要是水环境样品中污染物的富集和提取方面,涉及的污染物有工业废水以及生活污水中所带的有机污染物、重金属离子、固体悬浮物、难容或者不溶于水的悬浮液滴等。Lasarte-Aragonés等^[11]^将EAM与超高效液相色谱(UPLC)-DAD技术结合用于河流、自来水和水井3种水样中9种三嗪类除草剂的测定。所有样品中目标物的绝对回收率为48%~76%,相对回收率均接近100%,相对标准偏差RSD低于<9.3%。Yang等^[33]^构建了泡腾辅助注射器内分散固相微萃取技术与HPLC-UV联用用于富集和检测水中的拟除虫菊酯。在优化条件下,获得了良好的重复性(RSD, 1.7%~2.3%)、线性范围(2.5~500 μg/L)、检出限(LOD, 0.15~1.03 μg/L)和回收率(76.8%~86.5%),该方法成功地应用于河水、水库水和湖水等环境样品的检测。Fahimirad等^[22]^将EAM与微采样火焰原子吸收法(μS-FAAs)联用,成功检测了制药废水样品中的Pb(Ⅱ)、Cd(Ⅱ)、Ni(Ⅱ)和Cu(Ⅱ),富集因子高达100。Zhao等^[44]^采用基于磁性Fe_3_O_4_@多孔活性炭吸附剂的泡腾辅助深共晶熔剂分散液-液微萃取(META-DES-DLLME)结合HPLC方法成功应用于环境水中酚类内分泌干扰物的提取和分析。在优化的条件下,提取回收率为81.0%~94.7%, RSD为1.2%~6.7%, LOD和LOQ分别为0.82~1.7 μg/L和2.7~5.5 μg/L。Yang等^[37]^将EAM技术结合HPLC-UV用来检测4种杀菌剂(偶氮菌酯、三唑酮、赛普罗地尼、三氟菌酯),在最佳条件下,纯水模型和真实水样中的所有分析物均具有良好的线性。Shishov等^[72]^开发了一种用于水样现场预处理的泡腾片辅助可切换溶剂微萃取(ETA-SHS-ME)策略,通过HPLC-UV测定水样中的甾体激素(睾酮、孕酮、雌二醇和氢化可的松)。睾酮的线性范围为5~500 ng/L,孕酮的线性范围为25~750 ng/L,雌二醇的线性范围为10~1000 ng/L,氢化可的松的线性范围为50~500 ng/L。Xu等^[65]^建立了一种简单、高效、环保的磁泡腾辅助切换亲水性溶剂基液相微萃取-HPLC-DAD测定水样中三嗪类除草剂的方法。在50~5000 ng/mL范围内线性良好,相关系数大于0.997,检出限为0.10~0.13 ng/mL,在井水、池塘和河水中的提取回收率为81.4%~96.7%。

### 5.3 生物样品

在生物样品中,EAM技术已成功用于植物样品和动物的血样尿样中目标物的预浓缩与富集。众所周知,血样与尿样基质中含有大量的蛋白质分子、DNA、RNA、可溶性有机物、无机盐等,成分复杂,基质效应明显^[73]^。此外,目标物含量低,稳定性差,因此需要快速且高效的萃取方法。当样品黏度过大或者浓度过大,直接进行EAM处理,易造成吸附剂堵塞,甚至会加重基质效应,因此在进行EAM处理前,要对样品进行稀释、调节pH、去除大分子蛋白质等实际样品的前处理。将EAM技术用于经过前处理的样品可得到令人满意的萃取效果。Wang等^[24]^建立了一种基于碳纳米管-聚合物复合微球的泡腾辅助移液管尖端固相微萃取技术,用于提取淫羊藿生物样品中4种生物碱(木兰花碱、小檗碱、巴马定、麻根碱)和4种黄酮类物质(淫羊藿素A/B/C和淫羊藿素)。在萃取过程中,通过原位生成CO_2_,实现了mg级吸附剂的分散。在最佳分析条件下,线性范围为3~300 μg/L, RSD < 5%, LOD为1.02~2.98 μg/L,加标回收率为90.05%~99.85%。Jmshidi等^[18]^将EAM技术用于血浆中受体阻滞剂的富集。以布比卡因为内标,采用HPLC-MS检测人血浆中普萘洛尔、美托洛尔、阿替洛尔和阿普萘洛尔的含量,检出限分别为0.33、0.62、0.03和0.44 ng/mL。所有分析物均具有良好的线性关系,日内(*n*=5)和日间(*n*=10)的精密度均在6.3%以下,预浓缩因子在15~18范围内,萃取效率为75%~91%。Wu等^[13]^将泡腾辅助技术与离子液体原位转移反应相结合构建了基于ILs原位复分解反应的磁性泡腾辅助微萃取(ISMETA-ILDM)方法用于检测人尿液、孕妇血液和胎儿脐带血中4种内源性类固醇。磁性泡腾片由Fe_3_O_4_纳米颗粒、Na_2_CO_3_和TTA组成,泡腾反应过程中,NH_4_PF_6_与[C_6_MIM]BF_4_发生原位复分解,将亲水性离子液体变为疏水性离子液体,进而在水相中分离。与传统的ILs-DLLME方法相比,泡腾分散与磁性纳米颗粒相结合使得萃取剂的分散和收集几乎可以同时完成;与温控离子液体分散微萃取和冷诱导固化微萃取相比,该方法避免了加热和冷却过程,大大减少了时间和能量成本。Shishov等^[72]^结合泡腾反应和深共熔溶剂构建泡腾辅助DLLME法用于分离和预富集牛肝样品中的可电离酮洛芬和双氯芬酸2种非甾体抗炎药。该方法的过程包括两个阶段,首先,将肝脏样本置于pH=11的Na_2_CO_3_溶液中,利用目标物在碱性条件下的电离,促使其从固体样品相转移到碱性水相;其次,在含有目标物的碱性水相中加入由薄荷脑和甲酸(1∶40,物质的量比)组成的深共熔溶剂作为萃取溶剂,促使电离后的目标物由碱性水相转移至萃取溶剂中,以实现目标物的高效提取。该方法对酮洛芬和双氯芬酸的萃取效果良好,LOD分别为0.1和0.3 μg/kg。

## 6 展望

本文详细介绍了EAM技术方法的构建、萃取条件的优化、各类吸附剂及应用,分析了影响萃取效率的主要影响因素,介绍了该方法近年来在食品、环境和生物样品中目标物富集和检测中的应用进展。EAM技术作为新型快速萃取技术,操作简便、适用范围广,利用含有磁性的纳米材料、离子液体等新型吸附剂,提高了其萃取效率。经EAM技术或与EAM联用的各种技术进行预处理后的样品,可以直接使用液相色谱、气相色谱、原子吸收等大型仪器设备进行检测,使得EAM的应用范围更加广泛。进一步开发新型的吸附剂材料、优化其使用条件是促进该方法实现绿色化、小型化和现场化的重要手段。将该方法可联用的仪器进一步扩展到红外光谱、拉曼光谱等现场便携式仪器,进一步丰富前处理的方法及检测方法也是一个需要努力的方向。EAM技术同样存在一些局限性,因为需要CO_2_供体和H^+^供体的存在,使得吸附剂需具有一定的耐酸碱性质;浓度高或黏性大的样品如未经预处理,会造成吸附剂堵塞影响吸附效率;磁性吸附剂在用外部磁铁回收时,仍旧会有损失,影响其循环使用次数,这些问题仍需研究者们继续解决。此外,EAM技术的发展在很大程度上依赖于吸附剂的发展,因此需不断开发新型吸附剂,使得EAM技术更加完善。
